# Gamification in medical education: identifying and prioritizing key elements through Delphi method

**DOI:** 10.1080/10872981.2024.2302231

**Published:** 2024-01-09

**Authors:** Yung-Fu Wang, Ya-Fang Hsu, Kwo-Ting Fang, Liang-Tseng Kuo

**Affiliations:** a Department of Information Management, National Yunlin University of Science and Technology, Yunlin, Taiwan; b Department of Long-term Care and Health Promotion, Min-Hwei Junior College of Health Care Management, Tainan, Taiwan; c Division of Sports Medicine, Department of Orthopaedic Surgery, Chang Gung Memorial Hospital, Chiayi, Taiwan; d School of Medicine, Chang Gung University, Taoyuan, Taiwan

**Keywords:** Gamification, game-based learning, medical education, health professions education, Delphi method

## Abstract

**Background:**

Gamification has gained popularity in medical education, but key elements have not been formally identified. This study aimed to generate and prioritize a list of key elements of gamification in medical education.

**Methods:**

This study utilized a two-stage approach, including the Delphi method and qualitative interview. Nineteen medical educators with expertise in gamification participated in the Delphi method stage. Experts who had more than three years of experience with gamification in medical education constituted the expert panel. The experts were then asked to rate the gamification elements using the Likert five-point scale through at least two consensus-seeking rounds. Consensus for key elements was predefined as ≥ 51% of respondents rating an element as ‘important’ or”very important.” In the qualitative interview stage, 10 experts provided feedback on the application of these key gamification elements.

**Results:**

Eighteen participants (11 males and 7 females) completed the entire Delphi process for this study. After two rounds of surveys, the consensus was reached on all elements. Thirteen elements scored more than 4 points (37%) and reached the criteria of key elements of gamification in medical education. The top five key elements were integration with instruction objectives, game rules, rapid feedback, fairness, and points/scoring. The thirteen key elements for successful gamification in medical education were further organized into two main categories: (1) gamification design principles and (2) game mechanisms.

**Conclusions:**

Integration with educational objectives, gamification in curriculum design and teaching methods, and balancing between the mechanisms and principles were the three key components for successful gamification. This study explored the gamification key elements, providing practical tips for medical educators in their efforts to gamify medical education. Future studies involving learners could be performed to examine the efficacy of these key elements in gamification.

## Introduction

Gamification, defined as ‘the use of game design elements in non-game contexts’ [1–3], has been gaining popularity after 2010 [4–6]. Rather than playing games in the classroom, gamification is the insertion of gaming elements into an existing teaching program [7]. It is an educational design process and not a type of teaching method [7]. The students learn from the educational content but not from gamification [8]. Gamification of learning is the process of applying game elements to modify training content and methodology [8] to help develop student potential and provide positive encouragement to boost their performance level [9]. Implementing gamification in learning may elevate students’ interest and motivation to participate in the subject content [7,10], increase their concentration and enjoyment of learning [11], and help them with effective learning. The effects of gamification elements on learning are illustrated by both extrinsic motivation and intrinsic motivation [12], supported by the operant conditioning theory [3], the expectancy-based theories, the self-regulation [13], and the self-determination theories [8,14]. Gamification has been widely used in different fields such as business operations, marketing, education [15–17], and medical education [18–21].

Previous studies have identified several components of gamification [21,22]. Common gamification elements include points [23], badges [24], leaderboards [20], which can be used either individually or in combination [19,25]. Additional aspects commonly integrated in gamification implementation are progress bars, ranks, rewards, or incentives [26], along with the incorporation of a story or narrative. Further detailing, the design principles of gamification encompass include goals and challenges, personalization, rapid feedback, visual feedback, freedom of choice, freedom to fail, and social engagement [15]. Majuri et al. have expanded on these components by categorizing gamification elements into five types: progressive achievement, social interaction, immersion experience, non-digital elements, and others, noting that the most prevalent elements are points, challenges, badges, and a leaderboard [27]. Additionally, these key elements of gamification can be broadly classified into two categories, as suggested by the studies of Deterding et al. [4] and Dicheva et al. [15]: gamification design principles and game mechanism. This categorization helps in understanding the diverse yet interconnected nature of gamification components.

The growing literature on gamification in medical education shows promising learning outcomes by strengthening learning behaviors and attitudes [22]. However, debates still exist as the essential elements remain uncertain [7,28]. We are uncertain not only about what is important for gamification but also whether any specific element is effective. The leaderboard and the badges may be associated with discontentment for fairness [7] or have adverse impacts on learners already with intrinsic motivation [29]. The badge was perceived as the least motivating element [19], improving only motivated learners within the context of self-determination theory [29]. Competition can also be fatiguing, and the longevity of competition may be rugged [19,20]. A recent systematic review on gamification in medical education still could not recommend when and how specific elements should be applied with the existing limited evidence [22].

Furthermore, research related to educational gamification is still in the early stages [30]. In our prior research, the focus was on identifying critical elements of successful gamification in corporate training contexts [31]. Recognizing the distinct educational requirements of medical students, who must assimilate both theoretical knowledge and practical skills, we see a special applicability of gamification in medical education. In this study, we explored the unique role of gamification in medical education, acknowledging the specific needs of medical students for both theoretical and practical learning. We aim to offer expert opinion to support future implementing and study of gamification in medical education, and thus fill the gap in knowledge caused by thin and disconnected studies of gamification.

## Methods

### Study design

**Figure 1.**
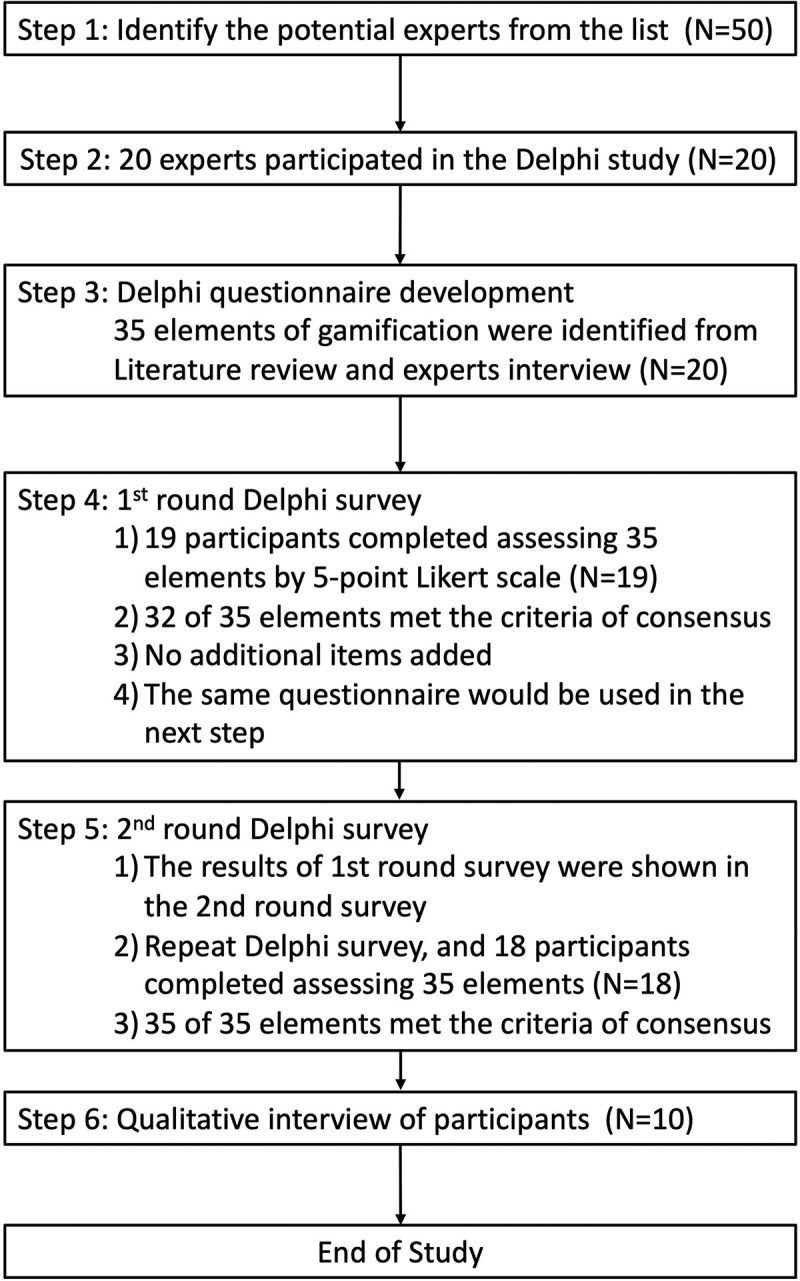
Study process.

This study was conducted in two stages from February to March 2021. In the first stage (Delphi stage), the gamification questionnaire was used to obtain consensus of medical educators on key elements of gamification in medical education. The questionnaire items used in this study were developed by combining literature review and expert input. These items had been previously utilized and validated in our previous research [31]. But a section had been added for respondents to express their own opinions (see Table 1 and Appendix 1). These responses were collected for use in the qualitative interviews. In the second stage (interview stage), a qualitative interview was conducted with each medical educators about their opinions and experiences in the application of key elements in the gamification. The Delphi technique means building consensus by administering a series of surveys to panelists with expertise within a particular area [32]. To reach consensus among the experts and select the key elements of gamification, this study conducted at least two rounds of expert-opinion collection, and the Delphi research stopped when consensus was reached [33]. The study process is shown in Figure 1. This study has been approved by the Institutional Review Boards of the Chang Gung Memorial Hospital (CGMH IRB 202100325B1).

**Table 1.** The description of the elements which used in the gamification questionnaire.

Elements	Description
Integration with training goal	Each activity designed for training gamification should be closely integrated with the training goals for the purpose of learning, not for fun or playing games.
Game rules	Clear game rules should be set up during the gamification process for the learners to follow.
Rapid feedback	In response to the learner’s performance, set up a rapid feedback mechanism and offer points, praises, chips, or even material rewards. The key is to provide feedback in a timely manner.
Fairness	Fairness is very important in training gamification, when points and rewards are provided by the teacher.
Points or scoring	A scoring mechanism is set up during the training gamification process, giving points to learners depending on their performance. For example, 1000 points are given for answering questions, and 5000 points are rewarded for completing group tasks, etc.
Team work	Teamwork is the basis of training gamification, requiring everyone to work together towards the same goals.
Competition	Design competitive activities as part of training gamification, prompting learners to compete individually or as a team.
Time Pressure	Set up time limits, prompting learners to complete assigned tasks quickly under time pressure.
Increasing difficulty	As part of training gamification, design the learner’s tasks with increasing levels of difficulty.
Freedom to fail	As part of training gamification, create an environment tolerant of mistakes or failure so the learners feel at ease and motivated to learn from their mistakes.
Task with challenging goals	As part of training gamification, design some difficult questions, tasks, or challenging goals that may be answered or completed only after hard work by the learners.
Prize or bonus money	Provide incentives, such as material rewards or cash prizes, to encourage learner participation.
Leaderboard	Display the learner or team performance ranking order to show everyone their own status relative to others in the training class.
Adaptation to difficulty	Make timely adjustments of the game or task difficulty based on each learner’s performance, which may require the use of a software program. Adjust the level of difficulty in any direction, not just from simple to complex.
Experiential activities	As part of training gamification, design various activities suitable for learner participation as well as post-activity evaluation, so learners may learn from the experience. For example, learners may learn the value of teamwork or project management via the cup-stacking activity, or learn to differentiate consultation vs. actual implementation through the activity of walking with a blindfold.
Educational tools or props	As part of training gamification, prepare teaching tools or props to facilitate the implementation of the training course.
Performance status feedback	Provide an index, a number, or some graphics to indicate the learner’s performance status.
Virtual currency or chips	Use coins, pretend-money or chips as scoring tools of training gamification.
Peer rating	Invite learners to evaluate each other as part of the learning process of gamified education.
Storyline	Use a story, background, or dialogues among different players during gamified training to help learners become immersed in the storyline.
Clues	Design a scavenger hunt as part of the gamified training, where the learners need to find a series of clues to discover the instructions for the next set of actions.
Virtual Helper	For training gamification, provide a real or virtual educational helper to answer questions or provide assistance to the learners.
Puzzles	During training gamification, design puzzles or problems for the learners to find the solutions. For example, create a jigsaw puzzle for the learners to solve.
Social network	Establish a channel of communication or social networking mechanism online or offline, so the learners may exchange and connect with each other. For example, set up a physical or virtual social group, or create a group discussion platform, etc.
Progress bar	As part of training gamification, create a status bar display to indicate the learner’s current progress in terms of the percentage of task completion or game progress.
Badges	Reward learners with different badges depending on their performance level, such as badges of gold, silver, bronze, platinum, or diamond. Badges are often given in place of prizes, to indicate hard work or accumulation of achievement in a game in class.
Customization or personalization	Offer a customizable training experience where the learners are able to make their own personalized adjustments and choose their own training content.
Board games	Use board games or commercial video games as part of the gamified training course or experiential activities.
Hierarchy	As part of gamified training, divide learners into different levels corresponding to different evolutions, such that they would obtain different capabilities after advancing to the next level. For example, *Pokémon Trainers* are divided into 1 ~ 40 levels. After a Trainer accumulates enough Experience Points, they can advance to the next level. Trainers unlock certain Features after they advance to the next level.
Virtual identity	Create a virtual identity or character for each learner in gamified training so they can play different roles, such as a magician, hunter, or animal, etc.
Integration with software apps	Use apps, relevant software, or websites to reach the goals of training gamification.
Virtual treasure	Learners are required to collect virtual treasures in the gamified training course, such as treasures or props in Pokémon.
Virtual reality	Use a 2D or 3D space simulation, virtual reality, or a game world situational simulation as part of the gamified training.
Health score	Assign a virtual health index corresponding to the character played by the learners during gamified training. When the health index reaches zero, it indicates that the character played by the learner needs to perform some task or obtain some material supplements.
Check in	Integrate GPS functionalities with the gamified training course, so the learners may mark their current location or perform a check in.

### Participants

While the number of panels may vary substantially, most studies using the Delphi technique involve around 15 to 20 experts [34]. Twenty experts were enrolled in this study, after recruiting health education teachers who were also healthcare professionals in the fields of medicine, pharmacy, nursing, physiology, and nutrition. The invited participants had met all the following criteria: (a) They had utilized common gamification elements such as points, scoring, and leaderboards within their educational courses. (b) They had had more than three years of experience with gamification in medical education. (c) They had been a lecturer at a medical college or teaching hospital in Taiwan over the past year.

Two gamification teaching experts served as the nominating team for this study. Previously, both experts had published textbooks and served as lecturers in the field of medical education. We identified 50 experts who had incorporated gamification in their teaching, and came from diverse types of medical education institutions, including medical schools, medical technology colleges, hospitals, and others. These experts were either recommended by peers, self-nominated, or known to us through our direct experience with their courses. From the list, invitations were extended to 30 potential participants via phone or email, and 20 experts agreed to participate in the research, though two of them withdrew in the middle of the process. The enrollment process is also shown in Figure 1. A total of 18 experts completed all two rounds of questionnaires, including 11 males (61.1%) and seven females (38.9%). The experts had a mean of 10.65 years of teaching experience and 3.71 years of gamification experience. The content of courses taught by these experts included the following fields: medicine (including internal medicine, orthopaedics, nephrology, emergency medicine, and CPR), evidence-based medical care, physiology, nutrition, pharmacy, evidence-based medicine, medical presentation skills, and communication skills. For detailed information on the experts who participated in this research, please refer to Table 2.

**Table 2.** Characteristics of study participants (*N* = 19).

Code	Gender	Profession	Years of Teaching Experience	Years of Gamification Experience	Fields of Educational Expertise
A	Male	Orthopaedist	10	3	Evidence-Based Medicine
B	Female	Occupational Therapist	7	3	Clinical Practice of Occupational Therapy
C	Male	Emergency Physician	10	3	Medical Care Act
D	Male	Orthopaedist	10	5	Orthopaedics
E	Female	Medical lecturer	21	4	Human Physiology
F	Female	Medical lecturer	12	4	Nutriology
G	Female	Nurse	4	3	Evidence-Based Medicine
H	Female	Case Manager	8	4	Doctor-Patient Communication
I	Male	Family Physician	10	5	Geriatrics
J	Male	Emergency Physician	9	3	Emergency Medicine
K	Male	Pulmonologist	8	3	Thoracic Medicine
L	Male	Pulmonologist	7	4	Interpret of Chest X-ray
M	Female	Dentist	10	3	Dentistry
N	Male	Oral and Maxillofacial Surgeon	18	6	Clinical Practice of Dentistry
O	Female	Nurse	10	4	Home Health Care
P	Male	Physiotherapist	15	3	Clinical Practice of Physical Therapy
Q	Male	Nurse	10	3	Cardiopulmonary Resuscitation
R	Male	Nephrologist	12	5	Clinical Practice of Nephrology
S^†^	Male	Emergency Physician	10	7	Emergency Medicine

^†^indicated the participant withdrew at the second round period.

### Two rounds of the Delphi process

For the first round, the experts were asked via email to evaluate the 35 elements of the gamification questionnaire (see Appendix 1) using the Likert five-point scale, where 5 was crucial and very important, 4 was important, 3 was a neutral, 2 was less important and 1 was very unimportant. The order of each expert survey question had been randomized to avoid ordering bias [35]. After completing the questionnaire, the experts were asked to write down additional key elements that had not been mentioned in the gamification questionnaire. In this study, the consensus criteria were established as inter-quartile range (IQR) ≤ 1 and standard deviation (SD) ≤1.5 [33], which are regarded as the requirements for a consensus of expert opinions. After a consensus was reached (a mean score ≥ 4, and more than 51% of the expert assessed as four or above), it was considered a gamification key element.

The Delphi process required the same questionnaire to be used for at least two rounds to obtain the experts’ consensus [36]. In the second round, experts were asked again to evaluate the 35 gamification elements and additional key elements provided by the experts in the first round. In addition to the questionnaire, the results of the first round were also presented, allowing the experts to consider the opinions of other participants and to modify their own opinion [36]. If the opinion of one expert exceeded the upper or lower IQR, that expert would be asked to provide an explanation. Descriptive statistics were then used to report the basic characteristics data of the participants, the percentages for each level of agreement for each element, the mean, SD, IQR, and the rankings using the agreement ratings [37]. All analyses were conducted via the SPSS 19 software.

### Qualitative interview

After the experts completed two rounds of surveys and reached a consensus, this study invited all 18 experts for interviews. They were asked for a detailed explanation of frequently used key elements of teaching gamification that they had listed on the questionnaire. Due to the COVID-19 pandemic, each expert was invited to an online conference that would last approximately 30–60 minutes, and 10 had accepted the invitation. The interview content was fully recorded and transcribed verbatim, with key points extracted to supplement the explanation of gamification elements.

## Results

Nineteen participants completed the first round of questionnaires, where 32 elements met the criteria of consensus in terms of IQR, SD, and mean value, and three elements did not reach consensus. Also, no additional key element items were introduced by the participants. Among the 32 elements, 16 elements scored more than 4 (45.71%), 12 elements scored between 3.0 to 3.9 (34.29%), and 7 elements scored less than 3.0 (20.0%). Since the Delphi method requires at least two rounds of surveys, all gamification elements evaluated were kept for a second round. Eighteen participants completed the second round of questionnaires, and one expert participating in the first round did not reply to the second round of questionnaires. After two rounds of the Delphi survey, the experts reached a consensus on all key elements. Thus, the Delphi survey was ended. Among the 35 elements, 13 scored more than 4 (37%), nine elements scored between 3.0 to 3.9 (26%), and the remaining 13 elements scored less than 3.0 (37%). Among the 13 elements with a score of greater than 4, more than 51% (ranging from 78% to 100%) of the experts assessed all these elements as ‘important’ or ‘very important.’ Therefore, these 13 items met the criteria of key elements of gamification in medical education. Table 3 shows the two rounds of the iterative process. The key elements of gamification were further divided into two categories based on the study of Deterding et al. [4] and Dicheva et al. [15]. Ten of thirteen were in the first category of gamification design principles, while the remaining three elements were in the second category of game mechanism.

**Table 3.** The results of two round-survey of experts’ opinion.

	Round 1					Round 2				
Elements	Mean	S.D.	IQR	≥4 Ratio	Rank	Mean	SD	IQR	≥4 Ratio	Rank
Integration with educational objectives	4.8	0.41	0	100%	1	4.9	0.23	0	100%	1
Game rules	4.8	0.41	0	100%	2	4.9	0.31	0	100%	2
Rapid feedback	4.8	0.41	0	100%	3	4.8	0.42	0	100%	3
Fairness	4.7	0.46	1	100%	5	4.7	0.47	1	100%	4
Points or scoring	4.7	0.44	0.5	100%	4	4.6	0.5	1	100%	5
Teamwork	4.7	0.57	0.5	95%	6	4.6	0.6	1	94%	6
Competition	4.5	0.68	1	89%	7	4.5	0.6	1	94%	7
Time Pressure	4.4	0.67	1	89%	12	4.4	0.68	1	89%	8
Increasing difficulty	4.4	0.59	1	95%	8	4.3	0.67	1	89%	9
Freedom to fail	4.3	0.86	1	84%	13	4.3	0.67	1	89%	9
Task with challenging goals	4.4	0.48	1	100%	11	4.2	0.60	0.75	89%	11
Prize or bonus money	4.1	0.72	1	79%	15	4.0	0.58	0	83%	12
Leaderboard	4.4	0.58	1	95%	11	4.0	0.67	0	78%	13
Adaptation to difficulty	4.1	0.72	1	79%	14	3.9	0.62	0	78%	14
Experiential activities	3.9	1	1.5*	74%	17	3.8	0.53	0.75	72%	15
Educational tools or props	4.4	0.88	1	84%	9	3.8	0.71	1	61%	16
Performance status feedback	4.1	0.91	1.5*	74%	16	3.7	0.56	1	67%	17
Virtual currency or chips	3.7	0.78	1	53%	18	3.4	0.83	1	56%	18
Peer rating	3.7	0.8	1	68%	19	3.4	0.83	1	50%	19
Storyline	3.5	0.75	1	58%	22	3.3	0.82	1	33%	20
Clues	3.6	0.88	1	42%	21	3.2	0.53	0.75	28%	21
Virtual Helper	3.6	0.75	1	53%	20	3.1	0.78	0	22%	22
Puzzles	3.4	0.67	1	47%	24	2.9	0.46	0	6%	23
Social network	3.5	1.19	1	53%	23	2.8	0.69	1	17%	24
Progress bar	3.4	1.04	1	37%	25	2.8	0.76	1	22%	25
Badges	3.3	0.78	1	37%	26	2.7	0.56	1	6%	26
Customization or personalization	3.2	0.89	1	37%	27	2.6	0.60	1	0%	27
Board games	2.8	0.74	1	21%	29	2.6	0.60	1	0%	28
Hierarchy	3.0	0.92	1	26%	28	2.6	0.68	1	0%	29
Virtual identity	2.8	1.06	1.5*	26%	30	2.4	0.76	1	6%	30
Integration with software apps	2.7	0.73	0.5	5%	31	2.4	0.76	1	0%	31
Virtual treasure	2.7	0.86	1	16%	32	2.2	0.69	1	0%	32
Virtual reality	2.5	0.82	1	11%	33	2.2	0.69	1	0%	33
Health score	2.5	0.88	1	5%	34	2.1	0.66	0.75	0%	34
Check in	2.4	0.94	1	11%^‡^	35	1.9	0.74	1	0%	35

Total 19 experts participated in the first round, while the 18 experts participated in the second round. At the first round, there 32 items met the criteria of consensus and 3 items did not meet. At the second round, all of the 35 items met the criteria of consensus.

*shown that did not meet the criteria of consensus.

SD: standard deviation; IQR: interquartile range; ≥ 4 Ratio: the numbers of 4 points and above/total numbers x 100%.

### Category I: gamification design principles

Gamification design principles are the foundational guidelines used to integrate game-like elements into non-gaming contexts. Based on previous studies, the following elements were categorized as gamification design principles, including setting the training goals and challenges, offering personalization options, providing feedback, ensuring freedom of choice and providing the opportunity to fail [15,31].

#### Integration with training goals

Quantitative data indicates a high prioritization of the element under study. It achieved an average score of 4.9 out of 5, demonstrating a consensus among the experts (IQR = 0, SD = 0.23). This uniformity in scoring is further underscored by 100% (18 out of 18) of the experts rating it as either ‘important’ or ‘very important’ (Table 3)

Qualitative analysis: gamification is not about playing games

‘What are the symptoms, and how severe are they? What are the necessary diagnostic criteria?… These are the key points. A related question will be formulated for each key point, whether it be a multiple-choice question or Q & A. The main points will be designed into the questions. It is not the case that the key points stayed in the past’. (Expert D)

‘The best, actually the better method is to have the educational goals first. Then, based on the game mechanics, different game mechanics, look for opportunities of integration’. (Expert K)

‘Set up your final teaching goals. Define quantitative teaching goals. If you can think clearly about such issues, then playing games for the sake of games can be avoided for the entire gamification process’. (Expert P)

Collectively, the experts concur that establishing clear educational objectives prior to curriculum design is vital. This ensures that the integration of gamification in medical education aligns with course objectives, thereby maximizing educational benefits [28].

#### Clear and fair game rules

Quantitative data highlights the significance of ‘Game rules’ and ‘fairness’ in the gamification of medical education. ‘Game rules’ ranked second with a mean score = 4.9 (IQR = 0, SD = 0.31), while ‘fairness’ ranked fourth, scoring an average of 4.7 (IQR = 1, SD = 0.47). These two elements were unanimously deemed either ‘important’ or ‘very important’ by all 18 experts (100%) involved in the study (Table 3).

Qualitative analysis: rules are the basic elements of game progress

Qualitative analysis showed that rules are the basic elements of game process [38]. Thus, the establishment of fairness in the gamification process, ensuring student participation and a fair rewarding system is an important gamification element.

‘How are teams divided at the very beginning? What are the prizes? How would it proceed, and how many parts are there? At the very beginning, we would go over the game rules’. (Expert R)

‘The syllabus itself is the contract we have with the students. Therefore, how the game rules are set up, we would explain these to them during the first week of class. The rules can also be considered as extra points or ways to get some extra points’. (Expert F)

‘They care very much. His eyes would glare at you. Did you give him the points? Also, for this question, why was I given 500 points. And then he and I answered the same way, but I did not get any points. This is something he cannot accept’. (Expert O)

Overall, the experts agree on the essential need to establish clear game rules with students, outlining team division, scoring methods, and operational aspects of the gamified curriculum. This foundation is crucial for smooth game proceedings. They also observed that increased student participation through gamification can lead to dissatisfaction if the game rules are perceived as unfair, emphasizing the need for equitable design and implementation of these rules [7].

#### Rapid feedback

Quantitative data places ‘Rapid feedback’ as the third most crucial element in the gamification of medical education, with a mean score of 4.8 (IQR = 0, SD = 0.42, Table 3). All experts assessed this element as either ‘important’ or ‘very important.’

Qualitative analysis: rapid feedback stimulates student response

Qualitative analysis showed that the provision of rapid feedback stimulated student response such that they were more willing to participate.

‘Regardless of whether the answer is right or wrong, if the teacher gives them a positive response or extra points as encouragement, students will be more motivated to answer questions next time’. (Expert P)

‘Provide feedback immediately; perhaps just give him some points … As long as there is an answer or some response, make sure there is feedback’. (Expert O)

The experts generally employed a combination of verbal and points-based feedback mechanisms. This approach is believed to foster a more engaging and responsive learning environment. Further elaboration on the implementation of these feedback mechanisms within the gamification framework will be provided in the subsequent section on gamification elements.

#### Team-based competition & challenging goals

Quantitative data shows the importance of ‘Teamwork’ and ‘Competition’ in gamified medical education. ‘Teamwork’ was ranked sixth with a mean score of 4.6 (IQR = 1, SD = 0.6), while ‘Competition’ followed closely, ranking seventh with a mean score of 4.5 (IQR = 1, SD = 0.6). Notably, 94% of experts (17 out of 18) assessed these two elements as either ‘important’ or ‘very important.’ Additionally, ‘Tasks with challenging goals’ emerged as another key element, ranking eleventh with a mean score of 4.2 (IQR = 0.75, SD = 0.6, Table 3).

Qualitative analysis: teams and competitions enhance participation

‘Working together with a team, he can minimize his elements. I am so dedicated because of the team. He feels a sense of honor and a sense of participation at the place’. (Expert J)

‘The main purpose of dividing into groups is for competition between the groups. The first goal is to increase motivation. The competition itself increases motivation. The second is that we have many clinics joining together … With the feeling of a team, there is greater cohesion’. (Expert P)

‘After gamification, there is a team score. They would find that he is very good at race-to-answer. He always puts his hand up quickly every time, as everyone has a common goal’. (Expert F)

The formation of teams in gamification not only integrates individual and team performance, but also helps alleviate individual fears of failure, thereby boosting student motivation, participation, and overall performance. The aspect of competition serves as a popular design principle in gamification, propelling participation and enthusiasm. Teachers may transform curriculum content into various contests and establish different challenging goals to motivate students to become engaged in the game. The experts concur that an appropriate application of gamification transforms the curriculum in a way that fosters significant learning outcomes through proactive student participation.

#### The degree of difficulty and pressure of gamification

The quantitative data highlights the importance of managing the degree of difficulty and pressure in gamification. ‘Time pressure’ is identified as a key element, ranking eighth with a mean score of 4.4 (IQR = 1, SD = 0.68). Similarly, both ‘Increasing difficulty’ and ‘Freedom to fail,’ are crucial, each ranking ninth with a mean score of 4.3 (IQR = 1, SD = 0.67). A significant majority of experts, 89% (16 out of 18), rated these three elements as either ‘important’ or ‘very important’ in gamification (Table 3).

Qualitative analysis: balancing difficulty with pressure

Qualitative analysis shows the importance of balancing the degree of difficulty and pressure of gamification while creating a risk-free environment for the participants. This is a key element considered very important by many experts.

‘Creating an interactive atmosphere is the most important. If it is difficult from the very beginning, of course, the students would be less willing to speak’. (Expert D)

‘When resources are lacking, such as when time is short, they can only work together to find a way out. Therefore, reducing the time serves two purposes for me: one is to focus their attention, and the other is forcing them to work together’. (Expert E)

“Points are given as long as a hand is raised regardless of right or wrong. Acknowledging their participation but not agreeing to their answer. Truly, I feel that this is something really important (Expert J)

The experts recommend starting with simple tasks in the initial stage of teaching gamification to facilitate easy participation. Balancing skills requirements and challenges optimizes classroom effectiveness. Time constraints are also deemed vital, as applying pressure can encourage teamwork and collective problem-solving. Crucially, a risk-free learning environment is advocated, where positive reinforcement is provided for participation, and there is no punishment for poor performance. This approach ensures that students have the ‘freedom to fail,’ encouraging them to participate confidently and without fear, even when their answers might be incorrect.

### Category II: game mechanism

Common gamification elements, as identified in prior research, include points, scoring systems, leaderboards, progress bars, ranks, and various forms of rewards or incentives [26,28,31]. These elements frequently feature in the implementation of gamification strategies.

#### Points

Quantitative data identifies ‘Points’ as a key element in the gamification of education. Ranking fifth, points scored a mean of 4.6 (IQR = 1, SD = 0.5, Table 3). A significant majority of experts, 94% (17 out of 18), considered this aspect ‘important’ or ‘very important.’

Qualitative analysis: points reflect student involvement

Qualitative analysis shows that a key element of teaching gamification is the provision of points, which serves as a tool to reflect the level of student involvement.

‘In the class, many questions are asked frequently, and point-accumulation starts … It may be an additional 100 or 200 points. If no one answers a question, I would just randomly add some points’. (Expert E)

‘After the groups are formed, I would keep the scores. The scoring method is to write some straight lines on the blackboard, so it is easy to add up. I count this score towards the grade at the end of the semester … I would use their performance during the race-to-answer session as the basis for extra points’. (Expert F)

‘We use playing cards. The way the cards are dealt feels great, and they think it is much fun. The cards are given as points, giving three, four, and five of the playing cards’. (Expert R)

The experts utilize various methods to integrate points into the curriculum, transforming content into interactive questions and activities. Points serve as the rapid feedback mechanism, significantly boosting student participation. Whether calculated on paper, a blackboard, or through creative means like playing cards, the use of points is seen as an effective motivator for students across different age groups and academic levels. This approach not only enhances participation but also positively impacts learning outcomes.

#### Prize or bonus money

The quantitative data identifies”Prize or bonus money” as an important gamification element, ranking it 12th with a mean score of 4.0 (IQR = 0, SD = 0.58, Table 3). A significant proportion of experts, 83% (15 out of 18), assessed this as ‘important’ or ‘very important.’

Qualitative analysis: prizes serve as badges of honor

‘When the laboratory class began, I showed the prize I was offering. What was special about the prize was that it had to be something that students liked … I would give them something they could not get on their own, and something that surprised them, that was my rat’s lung’. (Expert E)

‘When there are prizes, about 70% to 80% of the groups would want to raise their hands to answer the question first. Without prizes, students’ probability of raising their hands is about 30%, 40% to 50%. There is some difference in the degree of excitement’. (Expert C)

‘Prize – later on, I felt that it was more like letting him use it as a badge, to brag to others that he won the prize. It is not about putting it away for himself. It is that I did an excellent job, so I won this prize’. (Expert O)

The experts emphasize the strategic use of tangible or intangible prizes as incentives to boost student participation in educational gamification. They observe that the absence of prizes leads to a noticeable decline in student engagement. However, they also point out that the effectiveness of prizes goes beyond their material value; these rewards often carry symbolic significance, serving as emblems of achievement and participation that students can proudly showcase to their peers. This aspect of gamification, therefore, not only motivates students but also provides a sense of accomplishment and recognition within the educational setting.

#### Leaderboard

Quantitative data reveals the significance of the”Leaderboard” in gamification, ranking it 13th with a mean score of 4.0 (IQR = 0, SD = 0.67, Table 3). A considerable portion of experts, 78% (14 out of 18) rated the leaderboard as ‘important’ or ‘very important.’

Qualitative analysis: appropriate application of the leaderboard

‘I think the leaderboard is sometimes a double-edged sword … If the leaderboard can be tweaked so that everyone’s scores are close, the competition level would be better. However, if the gap is too big … the level of participation could decrease for the last few groups on the leaderboard’. (Expert L)

‘At every interval, everyone is allowed to look at the leaderboard. Sometimes it helps to motivate a little. Then, allow the groups at the bottom of the leaderboard the opportunity to get ahead, so that the groups in front would not get too relaxed’. (Expert R)

The leaderboard, by showing students their rankings compared to peers, inherently promotes competition and encourages contribution to study group. However, experts caution against potential demotivation among students in lower-ranked groups, especially if the point difference is substantial. This could lead to decreased participation or psychological discomfort.

There was some fluctuation in the experts’ assessment of the leaderboard’s importance. Some experts initially rated it as important (with 4 points), then revised it to unimportant (with 2 points), before ultimately reverting to considering it important during the qualification interview process. This shift underscores the complexity in effectively integrating a leaderboard into medical education gamification. The experts agreed that when applied thoughtfully, the leaderboard can be a valuable component of gamification strategy in medication education.

## Discussion

This study was the first in Asia to investigate the key elements of gamification in medical education. It identified thirteen key elements of gamification in medical education, categorized as the gamification design principles [4] and the game mechanics [15]. Our findings shown that in the category of gamification design principles, integration with course goals was the most important key element, followed by instant response, and the establishment of a gamified teaching environment, including team foundation, clear rules, and challenge goals. Furthermore, the common key elements of gamification in the mechanics category, points, and leaderboards, were considered effective motivators for learners only when used appropriately and in combination with other elements. A point system may be set up as the rapid feedback mechanism to strengthen student participation [7,39]. The leaderboard may be used to observe peer performance [28], which brings about competition [40], and continuously encourages the students to contribute toward their study group [7,11]. Although badges are sometimes regarded as rewards [28]. Our study has found that badges were not assigned significance or appropriately related to educational goals, and thus were not found to be a key element of gamification in medical education.

From the interviews portion of our study, these experts emphasized the importance of establishing game rules with learners as the basis for smooth progress, including how to group, how to score, and how the gamification course works, while being careful not to create an unfair or overly competitive environment. They also suggested utilizing team-building exercises and competitions to increase student engagement and reduce fear of failure. This is especially important in medical education, where mistakes can have serious consequences for patients. Gamification allows students to learn from their mistakes and improve their skills, without fear of failure. Through teamwork and collaboration in gamification, students learn how to communicate and collaborate effectively with other healthcare professionals, which is important to the future of medical education. However, the experts interviewed did not support the use of software or digital platforms as key gamification elements, nor did they support the reliance on board games and teaching props. Instead, they suggested that applying the key elements and mechanisms of gamification would be sufficient to redesign the curriculum utilizing educational gamification while enhancing student engagement and motivation.

### Implication for educators

The implementation of gamification in medical education presents some unique challenges. One such challenge is the potential for over-reliance on gamification elements, which could detract from the educational content and lead to reduced learning outcomes. To address this, educators must carefully consider the purpose and relevance of each gamification element and ensure they align with learning objectives and educational content. Furthermore, a lack of teacher support and training may impede the effective implementation of gamification in medical education. To help educators overcome these challenges, it is important to provide them with appropriate training in terms of the design and implementation of gamification elements, as well as access to relevant resources and technology to support their efforts. Moreover, it is crucial to consider the potential impact and side effects of gamification on the intrinsic motivation of students, which may be affected by extrinsic motivators such as gamification. Recent research has shown that intrinsic and extrinsic motivation can work together to predict performance, and a balanced approach that incorporates both types of motivation can be an effective way to promote student engagement and motivation [41]. Therefore, it is essential to strike a balance between intrinsic and extrinsic motivation while keeping the curriculum goals in mind.

Medical educators may also face challenges related to the ethical implications of gamification in medical education. For example, the use of rewards and incentives must be carefully considered to avoid potential conflicts of interest or inappropriate behavior. It is crucial to be cautious about the potential impact of gamification on students’ intrinsic motivation and moral responsibility to facilitate long-term student learning and professional development. These are some of the challenges medical educators may encounter when applying gamification in medical education.

To address these challenges, medical educators may choose from the following strategies. First, develop clear guidelines and policies for the use of gamification elements in medical education. The guidelines would include criteria for selecting appropriate gamification elements and formulating suitable implementation rules and procedures. Secondly, adequate support and training for effective gamification element design and implementation should be available to medical educators, including workshops, training classes, and access to resources and technology. Finally, the effectiveness of gamification elements and implementation methods should be continuously monitored and evaluated, followed by suitable modifications. This may involve tracking learning outcomes, gathering learner feedback, and assessing the ethical implications of gamification elements.

### Implications for future research

The findings of current study, the groundwork for future research in three critical areas: learner perspectives, the effectiveness of gamification elements, and learning outcomes in medical education. First, the study’s scope was limited to the identification of key gamification elements without incorporating the learners’ perspectives. Therefore, the opinions of the students on what is important in gamification may not be adequately represented. Future research should actively engage learners to understand their views on gamification’s essential aspects, thereby ensuring a more learner-centered approach. Such studies could offer deeper insights into how gamification elements resonate with learners and influence their educational experience. Secondly, while our study successfully identified key elements of gamification, it did not evaluate their impact on learning outcomes. Future research should focus on empirically assessing the efficacy of these elements in enhancing learning outcomes in medical education. This could involve experimental designs to measure the effectiveness of specific gamification strategies in improving knowledge retention, clinical skills, and learner engagement. Lastly, the current study did not measure the actual learning outcomes resulting from the application of gamification techniques. The relationship between gamification elements and learning efficacy remains an open question. It is essential for future longitudinal studies to track and evaluate the long-term effects of gamification on learning outcomes. Such studies could provide valuable data on the sustainability and impact of gamification strategies over time in medical education contexts.

### Limitations

Despite the insights gained, this study had certain limitations. One key limitation is the geographical focus; the research was conducted exclusively in Taiwan. This may restrict the generalizability of our findings to other educational systems which might have cultural and structural differences. These differences could impact educators’ experiences with gamification in various ways. Future research might need to encompass a broader range of countries and cultures to confirm and expand upon our current results.

## Conclusion

According to the results of this study, the crucial factors of successful gamification of medical education are: (a) closely following the educational objectives, (b) introduction of gamification into the curriculum design process and teaching methodology, and (c) giving equal importance to the game mechanics as well as game elements. This study explored the key elements and offers practical tips for medical educators in their efforts to gamify medical education. For future studies, the involvement of learners may be useful in examining these key elements of gamification with respect to learning efficacy.

## Supplementary Material

Appendix 1_20231221.docx

## Appendix 1

### Medical Teaching Gamification Questionnaire

Please, based on your real teaching experiences, thoughtfully select how important the following gamification elements are to your teaching. If there are other vital gamification elements not mentioned in this survey, feel free to add comments at the end.

This questionnaire lists 35 gamification elements. Please individually assess each element’s importance in your gamified teaching practice using a Likert five-point scale. Kindly differentiate between important and unimportant factors. The ratings are defined as follows:

**5: ‘Crucial and very important’**, indicating the element is almost always used in gamified teaching and is crucial for success.

**4: ‘Important’**, meaning the element is occasionally used in gamified teaching but still key for success.

**3: ‘Neutral value’**, suggesting the element is somewhat helpful in gamification and might have been used sparingly in the past.

**2: ‘Less Important’**, indicating the element has no impact on the success of gamified teaching.

**1: ‘Very Unimportant’**, suggesting the element might be misdefined and should not be considered a key element in teaching gamification.

**Table:**

Elements	Description	Scoring
Points or scoring	A scoring mechanism is set up during the training gamification process, giving points to learners depending on their performance. For example, 1000 points are given for answering questions, and 5000 points are rewarded for completing group tasks, etc.	
Leaderboard	Display the learner or team performance ranking order to show everyone their own status relative to others in the training class.	
Badges	Reward learners with different badges depending on their performance level, such as badges of gold, silver, bronze, platinum, or diamond. Badges are often given in place of prizes, to indicate hard work or accumulation of achievement in a game in class.	
Task with challenging goals	As part of training gamification, design some difficult questions, tasks, or challenging goals that may be answered or completed only after hard work by the learners.	
Hierarchy	As part of gamified training, divide learners into different levels corresponding to different evolutions, such that they would obtain different capabilities after advancing to the next level. For example, *Pokémon Trainers* are divided into 1~40 levels. After a Trainer accumulates enough Experience Points, they can advance to the next level. Trainers unlock certain Features after they advance to the next level.	
Team work	Teamwork is the basis of training gamification, requiring everyone to work together towards the same goals.	
Puzzles	During training gamification, design puzzles or problems for the learners to find the solutions. For example, create a jigsaw puzzle for the learners to solve.	
Progress bar	As part of training gamification, create a status bar display to indicate the learner’s current progress in terms of the percentage of task completion or game progress.	
Social network	Establish a channel of communication or social networking mechanism online or offline, so the learners may exchange and connect with each other. For example, set up a physical or virtual social group, or create a group discussion platform, etc.	
Performance status feedback	Provide an index, a number, or some graphics to indicate the learner’s performance status.	
Time Pressure	Set up time limits, prompting learners to complete assigned tasks quickly under time pressure.	
Storyline	Use a story, background, or dialogues among different players during gamified training to help learners become immersed in the storyline.	
Virtual identity	Create a virtual identity or character for each learner in gamified training so they can play different roles, such as a magician, hunter, or animal, etc.	
Competition	Design competitive activities as part of training gamification, prompting learners to compete individually or as a team.	
Virtual Helper	For training gamification, provide a real or virtual educational helper to answer questions or provide assistance to the learners.	
Health score	Assign a virtual health index corresponding to the character played by the learners during gamified training. When the health index reaches zero, it indicates that the character played by the learner needs to perform some task or obtain some material supplements.	
Increasing difficulty	As part of training gamification, design the learner’s tasks with increasing levels of difficulty.	
Peer rating	Invite learners to evaluate each other as part of the learning process of gamified education.	
Virtual currency or chips	Use coins, pretend-money or chips as scoring tools of training gamification.	
Virtual reality	Use a 2D or 3D space simulation, virtual reality, or a game world situational simulation as part of the gamified training.	
Board games	Use board games or commercial video games as part of the gamified training course or experiential activities.	
Customization or personalization	Offer a customizable training experience where the learners are able to make their own personalized adjustments and choose their own training content.	
Adaptation to difficulty	Make timely adjustments of the game or task difficulty based on each learner’s performance, which may require the use of a software program. Adjust the level of difficulty in any direction, not just from simple to complex.	
Prize or bonus money	Provide incentives, such as material rewards or cash prizes, to encourage learner participation.	
Game rules	Clear game rules should be set up during the gamification process for the learners to follow.	
Rapid feedback	In response to the learner’s performance, set up a rapid feedback mechanism and offer points, praises, chips, or even material rewards. The key is to provide feedback in a timely manner.	
Freedom to fail	As part of training gamification, create an environment tolerant of mistakes or failure so the learners feel at ease and motivated to learn from their mistakes.	
Experiential activities	As part of training gamification, design various activities suitable for learner participation as well as post-activity evaluation, so learners may learn from the experience. For example, learners may learn the value of teamwork or project management via the cup-stacking activity, or learn to differentiate consultation vs. actual implementation through the activity of walking with a blindfold.	
Integration with training goal	Each activity designed for training gamification should be closely integrated with the training goals for the purpose of learning, not for fun or playing games.	
Educational tools or props	As part of training gamification, prepare teaching tools or props to facilitate the implementation of the training course.	
Clues	Design a scavenger hunt as part of the gamified training, where the learners need to find a series of clues to discover the instructions for the next set of actions.	
Fairness	Fairness is very important in training gamification, when points and rewards are provided by the teacher.	
Integration with software apps	Use apps, relevant software, or websites to reach the goals of training gamification.	
Virtual treasure	Learners are required to collect virtual treasures in the gamified training course, such as treasures or props in Pokémon.	
Check in	Integrate GPS functionalities with the gamified training course, so the learners may mark their current location or perform a check in.	

Please add if there are any key gamification elements not included in the list above. Also, are there elements that you think require adjustments or additional descriptions?
