# Supplementary material for: Gamification in medical education: identifying and prioritizing key elements through Delphi method
Source: Med Educ Online. 2024 Jan 9;29(1):2302231. doi: 10.1080/10872981.2024.2302231 (PMC10778414; doi:10.1080/10872981.2024.2302231)
Supplement: Appendix 1_20231221.docx [file ZMEO_A_2302231_SM4065.docx]

# Appendix 1

# Medical Teaching Gamification Questionnaire

Please, based on your real teaching experiences, thoughtfully select how important the following gamification elements are to your teaching. If there are other vital gamification elements not mentioned in this survey, feel free to add comments at the end.

This questionnaire lists 35 gamification elements. Please individually assess each element's importance in your gamified teaching practice using a Likert five-point scale. Kindly differentiate between important and unimportant factors. The ratings are defined as follows:

**5: ' Crucial and very important '**, indicating the element is almost always used in gamified teaching and is crucial for success.

**4: 'Important'**, meaning the element is occasionally used in gamified teaching but still key for success.

**3: ' Neutral value '**, suggesting the element is somewhat helpful in gamification and might have been used sparingly in the past.

**2: 'Less Important'**, indicating the element has no impact on the success of gamified teaching.

**1: '****Very Unimportant'**, suggesting the element might be misdefined and should not be considered a key element in teaching gamification.

| **Elements** | **Description** | **Scoring** |
| --- | --- | --- |
| **Points or scoring** | A scoring mechanism is set up during the training gamification process, giving points to learners depending on their performance. For example, 1000 points are given for answering questions, and 5000 points are rewarded for completing group tasks, etc. |  |
| **Leaderboard** | Display the learner or team performance ranking order to show everyone their own status relative to others in the training class. |  |
| **Badges** | Reward learners with different badges depending on their performance level, such as badges of gold, silver, bronze, platinum, or diamond. Badges are often given in place of prizes, to indicate hard work or accumulation of achievement in a game in class. |  |
| **Task with challenging goals** | As part of training gamification, design some difficult questions, tasks, or challenging goals that may be answered or completed only after hard work by the learners. |  |
| **Hierarchy** | As part of gamified training, divide learners into different levels corresponding to different evolutions, such that they would obtain different capabilities after advancing to the next level. For example, *Pokémon* *Trainers* are divided into 1~40 levels. After a Trainer accumulates enough Experience Points, they can advance to the next level. Trainers unlock certain Features after they advance to the next level. |  |
| **Team work** | Teamwork is the basis of training gamification, requiring everyone to work together towards the same goals. |  |
| **Puzzles** | During training gamification, design puzzles or problems for the learners to find the solutions. For example, create a jigsaw puzzle for the learners to solve. |  |
| **Progress bar** | As part of training gamification, create a status bar display to indicate the learner’s current progress in terms of the percentage of task completion or game progress. |  |
| **Social network** | Establish a channel of communication or social networking mechanism online or offline, so the learners may exchange and connect with each other. For example, set up a physical or virtual social group, or create a group discussion platform, etc. |  |
| **Performance status feedback** | Provide an index, a number, or some graphics to indicate the learner’s performance status. |  |
| **Time Pressure** | Set up time limits, prompting learners to complete assigned tasks quickly under time pressure. |  |
| **Storyline** | Use a story, background, or dialogues among different players during gamified training to help learners become immersed in the storyline. |  |
| **Virtual identity** | Create a virtual identity or character for each learner in gamified training so they can play different roles, such as a magician, hunter, or animal, etc. |  |
| **Competition** | Design competitive activities as part of training gamification, prompting learners to compete individually or as a team. |  |
| **Virtual Helper** | For training gamification, provide a real or virtual educational helper to answer questions or provide assistance to the learners. |  |
| **Health score** | Assign a virtual health index corresponding to the character played by the learners during gamified training. When the health index reaches zero, it indicates that the character played by the learner needs to perform some task or obtain some material supplements. |  |
| **Increasing difficulty** | As part of training gamification, design the learner’s tasks with increasing levels of difficulty. |  |
| **Peer rating** | Invite learners to evaluate each other as part of the learning process of gamified education. |  |
| **Virtual currency or chips** | Use coins, pretend-money or chips as scoring tools of training gamification. |  |
| **Virtual reality** | Use a 2D or 3D space simulation, virtual reality, or a game world situational simulation as part of the gamified training. |  |
| **Board games** | Use board games or commercial video games as part of the gamified training course or experiential activities. |  |
| **Customization or personalization** | Offer a customizable training experience where the learners are able to make their own personalized adjustments and choose their own training content. |  |
| **Adaptation to difficulty** | Make timely adjustments of the game or task difficulty based on each learner’s performance, which may require the use of a software program. Adjust the level of difficulty in any direction, not just from simple to complex. |  |
| **Prize or bonus money** | Provide incentives, such as material rewards or cash prizes, to encourage learner participation. |  |
| **Game rules** | Clear game rules should be set up during the gamification process for the learners to follow. |  |
| **Rapid feedback** | In response to the learner’s performance, set up a rapid feedback mechanism and offer points, praises, chips, or even material rewards. The key is to provide feedback in a timely manner. |  |
| **Freedom to fail** | As part of training gamification, create an environment tolerant of mistakes or failure so the learners feel at ease and motivated to learn from their mistakes. |  |
| **Experiential activities** | As part of training gamification, design various activities suitable for learner participation as well as post-activity evaluation, so learners may learn from the experience. For example, learners may learn the value of teamwork or project management via the cup-stacking activity, or learn to differentiate consultation vs. actual implementation through the activity of walking with a blindfold. |  |
| **Integration with training goal** | Each activity designed for training gamification should be closely integrated with the training goals for the purpose of learning, not for fun or playing games. |  |
| **Educational tools or props** | As part of training gamification, prepare teaching tools or props to facilitate the implementation of the training course. |  |
| **Clues** | Design a scavenger hunt as part of the gamified training, where the learners need to find a series of clues to discover the instructions for the next set of actions. |  |
| **Fairness** | Fairness is very important in training gamification, when points and rewards are provided by the teacher. |  |
| **Integration with software apps** | Use apps, relevant software, or websites to reach the goals of training gamification. |  |
| **Virtual treasure** | Learners are required to collect virtual treasures in the gamified training course, such as treasures or props in Pokémon. |  |
| **Check in** | Integrate GPS functionalities with the gamified training course, so the learners may mark their current location or perform a check in. |  |
| **Please add if there are any key gamification elements not included in the list above. Also, are there elements that you think require adjustments or additional descriptions?** | | |
